# Umuhengerin Neuroprotective Effects in Streptozotocin-Induced Alzheimer’s Disease Mouse Model via Targeting Nrf2 and NF-Kβ Signaling Cascades

**DOI:** 10.3390/antiox10122011

**Published:** 2021-12-18

**Authors:** Alaa Sirwi, Nesrine S. El Sayed, Hossam M. Abdallah, Sabrin R. M. Ibrahim, Gamal A. Mohamed, Ali M. El-Halawany, Martin K. Safo, Nora O. Abdel Rasheed

**Affiliations:** 1 Department of Natural Products and Alternative Medicine, Faculty of Pharmacy, King Abdulaziz University, Jeddah 21442, Saudi Arabia; asirwi@kau.edu.sa (A.S.); gamals2001@yahoo.com (G.A.M.); 2 Department of Pharmacology and Toxicology, Faculty of Pharmacy, Cairo University, Giza 11562, Egypt; nesrine.salah@pharma.cu.edu.eg (N.S.E.S.); nora.osama@pharma.cu.edu.eg (N.O.A.R.); 3 Department of Pharmacognosy, Faculty of Pharmacy, Cairo University, Giza 11562, Egypt; ali.elhalawany@pharma.cu.edu.eg; 4 Preparatory Year Program, Department of Chemistry, Batterjee Medical College, Jeddah 21442, Saudi Arabia; sabrin.ibrahim@bmc.edu.sa; 5 Department of Pharmacognosy, Faculty of Pharmacy, Assiut University, Assiut 71526, Egypt; 6 Department of Pharmacognosy, Faculty of Pharmacy, Al-Azhar University, Assiut 71524, Egypt; 7 Department of Medicinal Chemistry, Institute for Structural Biology, Drug Discovery and Development, School of Pharmacy, Virginia Commonwealth University, Richmond, VA 23219, USA; msafo@vcu.edu

**Keywords:** umuhengerin, methoxy flavonoids, dementia, sporadic Alzheimer’s disease

## Abstract

Alzheimer’s disease (AD) is the most common type of dementia and is characterized by advanced cognitive deterioration, deposition of Aβ (amyloid-beta), and the formation of neurofibrillary tangles. Administration of streptozotocin (STZ) via the intracerebroventricular (ICV) route is a reliable model resembling sporadic AD (SAD) associated neuropathological changes. The present study was undertaken to explore the neuroprotective effects of the methoxy flavonoid, umuhengerin, in an STZ-induced SAD mouse model as a potential therapy for AD. Mice were injected once with STZ (3 mg/kg, ICV), followed by daily administration of umuhengerin (orally, 30 mg/kg) or the positive control donepezil (orally, 2.5 mg/kg) for 21 days. The pharmacological activity of umuhengerin was assessed through estimation of oxidative stress and inflammatory markers via mouse ELISA kits, Western blot analysis, and brain histopathological examination. Morris water maze test was also conducted to investigate umuhengerin-induced cognitive enhancement. The results showed that umuhengerin attenuated STZ-produced neuroinflammation and oxidative stress with a notable rise in the expression of Nrf2 (nuclear factor erythroid 2-related factor 2). In contrast, it downregulated Keap-1 (Kelch-like ECH associated protein 1), as well as elevated brain contents of GSH (reduced glutathione) and HO-1 (heme oxygenase-1). STZ-injected animals receiving umuhengerin showed marked downregulation of the nuclear factor kappa beta (NF-Kβp65) and noticeable increment in the expression of its inhibitor kappa beta alpha protein (IKβα), as well as prominent reduction in malondialdehyde (MDA), H_2_O_2_ (hydrogen peroxide), and TNF-α (tumor-necrosis factor-alpha) contents. Β-secretase protein expression and acetylcholinesterase (AchE) activity were also diminished upon umuhengerin injection in the STZ group, leading to decreased Aβ formation and cognitive improvement, respectively. In conclusion, umuhengerin neuroprotective effects were comparable to the standard drug donepezil; thus, it could be an alternative approach for AD management.

## 1. Introduction

Alzheimer’s disease (AD) is characterized by Aβ (amyloid-β) plaque accumulation and neurofibrillary tangles formation [1]. SAD (sporadic AD), which constitutes most AD cases, is associated with numerous pathological changes, such as cholinergic deficit, oxidative stress, and neuroinflammation, which result in tau hyperphosphorylation and deposition of Aβ in the brain [2]. Oxidative stress (OS) is an important factor contributing to AD pathogenesis, as it is linked to the formation of neurofibrillary tangles and Aβ plaques. It induces the formation of insoluble Aβ peptides and their accumulation. OS is also reported to reduce α-secretase activity while increasing that of β-secretase, which contributes to toxic Aβ formation [3]. OS, along with Aβ deposition, also provoke the generation of various inflammatory cytokines that are implicated in neuronal damage. TNF-α, a proinflammatory cytokine, was reported to induce NF-kβ (nuclear factor kappa beta) which in turn, promotes the further release of various proinflammatory cytokines leading to a vicious cycle of neuroinflammation [4]. Upon its activation, phosphorylated NF-kβ dimers are dissociated from the inhibitor kappa beta complex (IKβ) and translocated to the nucleus where they promote cytokines generation. Moreover, activated NF-kβ is implicated in β-secretase activation with subsequent Aβ deposition [5]. Thus, the inhibition of NF-kβ could retard AD [4].

The nuclear factor erythroid 2-related factor 2 (Nrf2) is involved in regulating several antioxidant response elements [6]. Nrf2 is normally located in the cytosol and linked to Keap-1 (Kelch-like ECH-associated protein 1). Under OS, Nrf2 detaches from Keap-1 and translocates into the nucleus where it binds to antioxidant response elements and regulates the expression of various stress proteins and detoxifying enzymes, such as heme oxygenase-1 (HO-1), which reduces ROS production [6]. Nrf2 also shows a neuroprotective effect by inducing postinjury hippocampal neurogenesis [7]. Additionally, Nrf2 inhibits the production of proinflammatory factors. Therefore, Nrf2 is thought to prevent neuronal death by combating oxidative stress and neuroinflammation, which accompany AD pathology [7]. Acetylcholinesterase (AChE), the enzyme involved in acetylcholine hydrolysis, has shown augmented activity around Aβ plaques, enhancing the cytotoxicity of Aβ peptides. Consequently, it was suggested that the AChE enzyme is linked to amyloid toxicity associated with AD. Thus, many of the FDA-approved drugs for AD treatment are cholinesterase inhibitors, with donepezil as an example. These drugs are able to reverse the decreased levels of acetylcholine neurotransmitters associated with AD pathology, which is expected to improve memory and learning functions through cholinergic transmission enhancement [1].

Injection of a small dose of streptozotocin (STZ) intracerebroventricularly (ICV) is reported to serve as a reputable mimicker model of SAD, as the injected animals displayed behavioral, neurochemical, and structural changes that accompany human SAD. Moreover, this model is associated with a marked increment in oxidative stress and proinflammatory markers, as well as cholinergic derangement, Aβ accumulation, and cognitive impairment, which are well-known pathological changes linked to SAD [8].

In recent years, regular intake of food rich in flavonoids has been linked with humans’ augmented cognitive abilities via the protection of neurons against oxidative stress, and the inhibition of AChE and β-secretase activities [9]. Moreover, these are shown to be capable of augmenting vascular blood flow and initiating neurogenesis [10]. *Psiadia punctulata* (PP), a plant mostly found in the tropics of Africa, Asia, and Saudi Arabia, is known to have diverse phytochemicals, such as flavonoids [11,12]. It is reported that 70% methanolic extract of PP has a blood pressure-lowering effect, which is suggested to be due to endothelial nitric oxide and Ca^2+^-dependent eNOS [11]. The vasodilator activity of PP on constricted vessels is also linked to its flavonoids’ protective effect on advanced glycation end products [12]. Umuhengerin, a pentamethoxy flavone, represents a major constituent of PP and is known for its beneficial effect against bacterial and fungal infections [13]. Due to flavonoid-associated positive pharmacologic effects, we undertook this study to investigate umuhengerin as a potential candidate for combating STZ-induced neurodegeneration and cognitive decline.

## 2. Materials and Methods

### 2.1. Isolation and Purification of Umuhengerin

Umuhengerin was isolated from aerial parts of *Psiadia punctulata,* following the published procedures [11,12]. The plant was collected from the west region in Saudi Arabia and identified by Dr. Emad Al-Sherif, Faculty of Science & Arts, Khulais, King Abdulaziz University, KSA. A specimen (PP-1065) was kept at the Herbarium of the Faculty of Pharmacy, King Abdulaziz University.

### 2.2. Animals

Swiss adult male albino mice (3–4 months, 25–30 g) were acquired from the animal house facility of the Faculty of Pharmacy Cairo University. The animals were free to access water and food. The investigation was approved (approval number: CU III F 27 20) by CU-IACUC (Institutional Animal Care & Use Committee) and guided by the policies of the NIH (National Institutes of Health) Guides for Care and Use of Laboratory Animals (2011).

### 2.3. Materials

Donepezil and streptozotocin (STZ) (Sigma-Aldrich, St. Louis, MO, USA) were dissolved in 0.9% saline [14,15].

### 2.4. SAD Induction

The freehand I.C.V. method by Pelleymounter et al. [16,17], and amended by Warnock [18], was used to avoid the penetration of the cerebral vein. The needle was injected at the following coordinates from bregma; mediolateral (1 mm), anteroposterior (−0.1 mm), and dorsoventral (−3 mm). Mice conducted normally 1 min subsequent to the injection [19].

### 2.5. Design of Experiment

Mice were categorized in a random manner into five groups (10 animals each). Group 1 (control group) received ICV saline (0.9%) as well as distilled water with 5% carboxymethylcellulose orally, whereas group 2 was composed of normal animals receiving umuhengerin (30 mg/kg/day, oral) [13]. Group 3 was given 3 mg/kg STZ (ICV) [20] once. Groups 4 and 5 were given 3 mg/kg STZ (ICV) once. Subsequently, after 5 h, group 4 received umuhengerin (30 mg/kg/day, oral), whereas group 5 was injected with donepezil (2.5 mg/kg/day, oral) [21]. Both umuhengerin and donepezil were injected daily for 21 days. Morris water maze test was conducted 24 h subsequent to the end of the injection plan.

### 2.6. Morris Water Maze (MWM) Test

The assessment was conducted as previously reported on five sequential days. The mean escape latency (MEL) during each performed trial over the first four days was recorded and considered as an acquisition index, and the time consumed in the target quadrant by each mouse where the hidden platform was previously placed was estimated and referred to as a measure of retrieval [22].

### 2.7. Tissue Sampling

After the MWM test was carried out, mice were euthanatized by cervical dislocation and decapitation. The brains were rapidly excised and washed in a salt/ice mixture, Afterwards, they were weighed and homogenized in saline to prepare 10% homogenates that were centrifuged. The supernatants were used for estimating hydrogen peroxide (H_2_O_2_), reduced glutathione (GSH), and malondialdehyde (MDA) brain contents. TNF-α and HO-1 contents, along with acetyl cholinesterase (AChE) enzyme activity, were also assessed.

### 2.8. Estimation of Biochemical Parameters

Mouse ELISA kits were used for the assessment of GSH, MDA, and H_2_O_2_ brain contents (My Bio Source, San Diego, CA, USA), as well as TNF-α, HO-1, and AChE enzyme (Cusabio, Wuhan, China).

### 2.9. Western Blot Analysis

The study was performed for estimating Nrf2, Keap-1, β-secretase, NF-Kβ-p65, as well as its inhibitor’s IKβα activities. The procedure was carried out as previously reported by [20] with the nitrocellulose membranes being incubated with 1:1000 dilutions of the following primary antibodies: Nrf2, β-secretase, NF-Kβ p65, Keap-1, and IKβα (Cell Signaling Technology, Danvers, MA, USA). Afterwards, they were probed with the peroxidase-labeled secondary antibodies (Dianova, Hamburg, Germany). Lastly, the band intensities were specified by densitometric analysis using a scanning laser densitometer. Results were displayed as arbitrary units relative to the corresponding β-actin band intensity.

### 2.10. Histopathological Examination

The brains of 3 animals were fixed for at least for 48 h in neutral buffered formalin (10%). Thereafter, washing, dehydration in alcohol, and embedding in paraffin blocks of brains were carried out. For preliminary histopathological examination, tissue sections with 4 μm thickness were stained with hematoxylin and eosin stain (H&E). Furthermore, Congo red stain was applied to reveal amyloid plaques according to [23] method. For the assessment of neuronal loss, the surviving neurons in the cerebral cortex and hippocampal regions; dentate gyrus (DG), and cornu ammonis (CA 3 and 4) were quantified as stated by [24]. The rate of neuronal survival was expressed as the intact neuron’s percentage. Nissl-stained sections of the cerebral cortex and the hippocampus (CA3, CA4, and DG) were used for the assessment of neurodegeneration [25].

### 2.11. Statistical Analysis

Analysis of the results was conducted by employing ANOVA (one-way analysis of variance) and Tukey’s multiple comparison test, respectively, and the results were represented as mean ± SEM, using GraphPad Prism software (VER 6.01; Graph Pad Software, San Diego, CA, USA). The mean escape latency was evaluated in MWM trials by repeated measures (ANOVA). The significance level was set at *p* < 0.05.

## 3. Results

### 3.1. Characterization of Isolated Compounds

**Figure 1:**
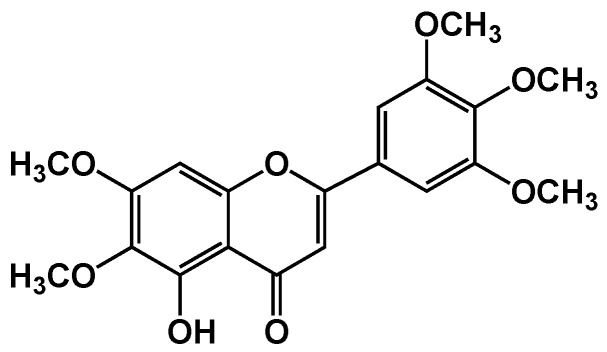
Chemical structure of Umuhengerin.

Umuhengerin (Figure 1) was isolated from Psiadia punctulate and identified based on their NMR data as previously published [11].

### 3.2. Effect of Umuhengerin on the Behavior of the Animals Receiving STZ during Morris Water Maze Task

**Figure 2:**
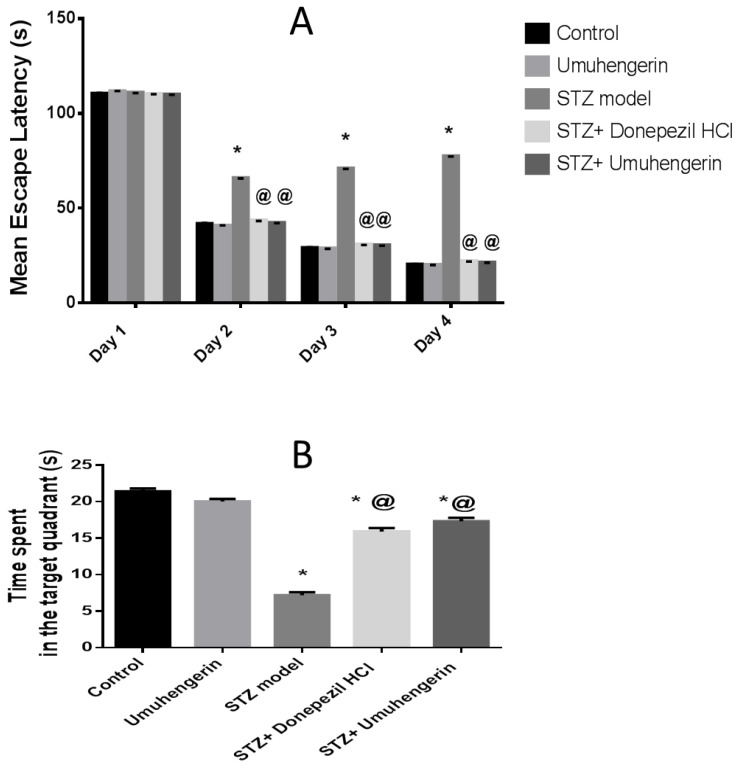
Effect of umuhengerin on the behavior of the animals receiving STZ during the Morris water maze test. (**A**) Mean escape latency (MEL) and (**B**) the time spent in the target quadrant. Values are expressed as mean ± SEM; *n* = 10. * *p* < 0.05 vs. control group, @ *p* < 0.05 vs. ICV-STZ group.

The animals started spending less time reaching the platform in the following 3 days, with the STZ group having the maximum mean escape latency (MEL). Umuhengerin administration to normal mice did not alter the MEL significantly. On the second, third, and fourth trial days, animals administered STZ alone showed a marked increase in the MEL, as compared with control animals. Treatment with either umuhengerin or donepezil showed significant amelioration in the MEL with no remarkable variation among the treated groups. As for the test day, normal animals that received umuhengerin showed no significant difference in the time spent in the target quadrant in which the platform was formerly placed, in comparison with control mice. An apparent decrease in the time consumed in the target quadrant was observed in the STZ group. Treated groups showed a noticeable increase in the time spent in the target quadrant, as compared with the STZ group with no obvious difference among umuhengerin and donepezil-treated groups. Accordingly, administration of umuhengerin to STZ-injected mice revealed significant progress in spatial memory, comparable to the standard drug donepezil (Figure 2).

### 3.3. Effect of Umuhengerin on Oxidative Stress Associated with STZ Administration

**Figure 3:**
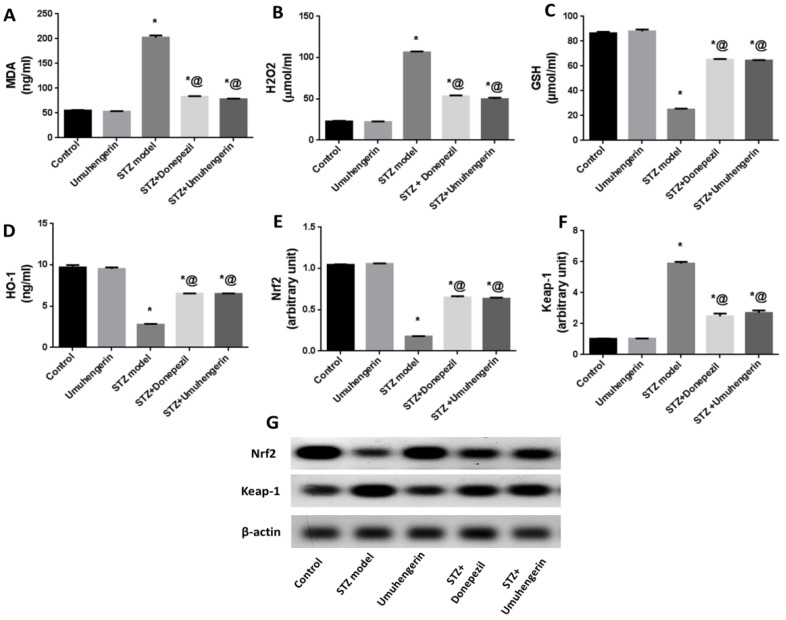
Effect of umuhengerin on oxidative stress associated with STZ administration. (**A**) MDA, (**B**) H_2_O_2_, (**C**) GSH, and (**D**) HO-1 brain contents were estimated, in addition to (**E**) Nrf2 and (**F**) Keap-1 protein expression. (**G**) Western blot of Nrf2 and Keap-1. Values are expressed as mean ± SEM; *n* = 7. * *p* < 0.05 vs. control group, @ *p* < 0.05 vs. ICV-STZ group.

The animals which received STZ injections showed a significant increase in oxidative stress that was reflected in reduced heme oxygenase (HO-1) and reduced glutathione (GSH) contents. In contrast, there were elevations of malondiadehyde (MDA) and hydrogen peroxide (H_2_O_2_) contents. Moreover, a prominent decrease in Nrf2 protein expression was observed in this group, whereas its associated protein, Keap-1, was upregulated when compared with the normal control group. Treatment with umuhengerin or donepezil abolished STZ-induced oxidative stress with increased GSH and HO-1 contents, as well as Nrf2 upregulation in contrast to decreased MDA and H_2_O_2_ contents and Keap-1 downregulation. Summarily, both umuhengerin and donepezil hindered STZ-induced oxidative damage. Administration of umuhengerin in normal mice showed no substantial difference in the above marks as compared with the normal control group (Figure 3).

### 3.4. Effect of Umuhengerin on Neuroinflammation Secondary to STZ Administration

**Figure 4:**
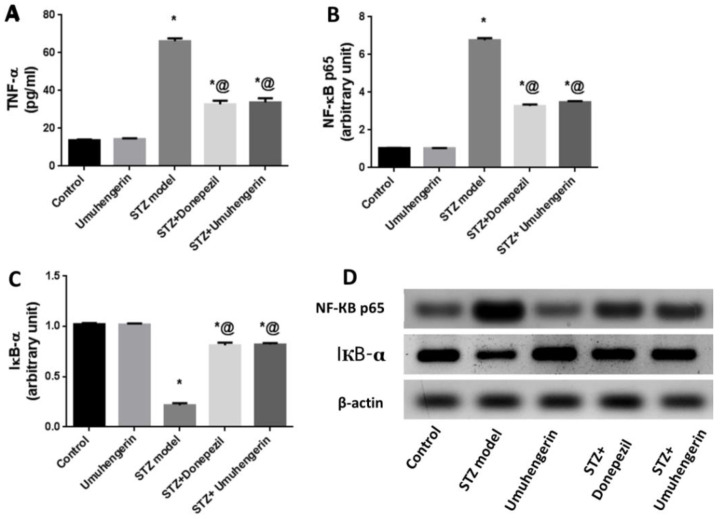
Effect of umuhengerin on neuroinflammation secondary to STZ administration. (**A**) TNF-α content as well as (**B**) NF-kβ p65, and (**C**) IKβα protein expression. (**D**) Western blot analysis of NF-kβ p65 and p-IKβα. Values are expressed as mean ± SEM; *n* = 7. * *p* < 0.05 vs. control group, @ *p* < 0.05 vs. ICV-STZ group.

Unlike control mice, STZ-induced mice showed a prominent induction of NF-Kβp65 protein expression with an obvious increase in TNF-α in the brain content, which contrasts with the noticeable downregulation of its inhibitor kappa beta alpha (IKβα) protein. Administration of umuhengerin or donepezil to STZ-induced mice ameliorated elevation of the proinflammatory markers linked to STZ injection, with reduced TNF-α content, along with inhibition of NF-Kβp65 and upregulation of the IKβα protein expression. Thus, umuhengerin, like donepezil, impeded STZ-induced neuroinflammation. There were no noticeable changes in the above-mentioned markers with the administration of umuhengerin to normal mice, when compared to the normal control group (Figure 4).

### 3.5. Effect of Umuhengerin on the STZ-Mediated Increase in AChE Enzyme Activity and β-Secretase Protein Expression

**Figure 5:**
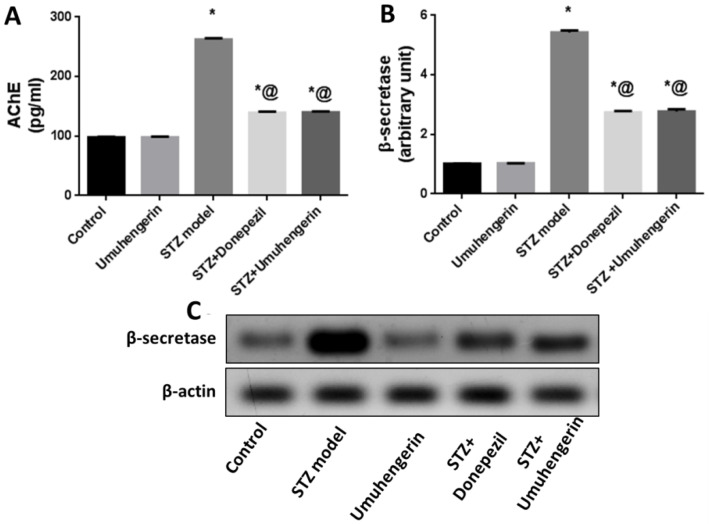
Effect of umuhengerin on an STZ-induced increase in (**A**) AChE activity and (**B**) β-secretase protein expression. (**C**) Western blot analysis of β-secretase. Values are expressed as mean ± SEM; *n* = 7. * *p* < 0.05 vs. control group, @ *p* < 0.05 vs. ICV-STZ group.

Administration of STZ has been implicated in the prominent elevation of acetylcholinesterase (AChE) enzyme activity and β-secretase protein expression. Treatment with either umuhengerin or donepezil combated STZ-induced activation of these two enzymes resulting in reduced Aβ deposition and enhanced cholinergic transmission. Thus, umuhengerin attenuated STZ-induced hazardous Aβ aggregation and cholinergic dysfunction in a comparable manner to the standard drug donepezil. Administration of umuhengerin in normal mice showed no considerable difference in the above markers when compared with the normal control group (Figure 5).

### 3.6. Effect of Umuhengerin Administration on Mice Brain Histopathological Alterations Owing to STZ Administration

**Figure 6:**
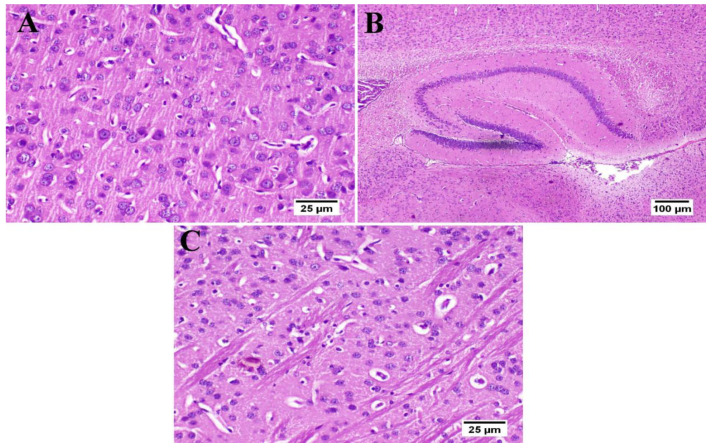
Histology of normal control mice brain (*n* = 3) (H&E). Normal control mice (group I) showed normal structure of different neurons in the (**A**) cerebral cortex, (**B**) hippocampus, and (**C**) striatum.

**Figure 7:**
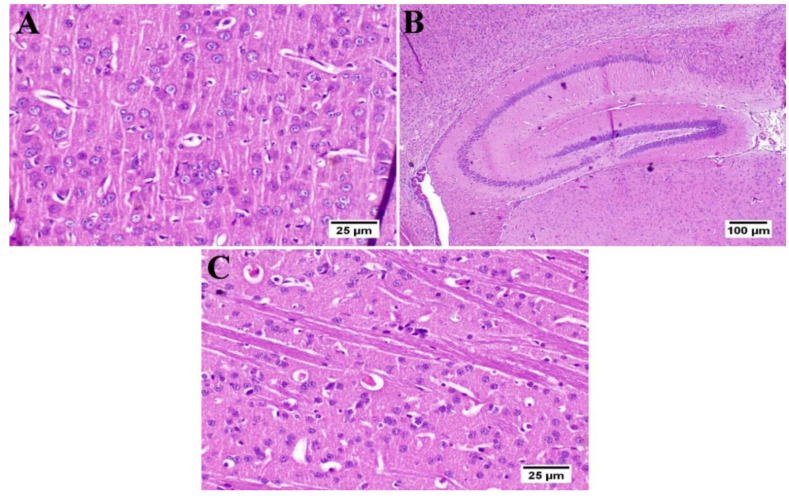
Histology of the brain of normal mice which received umuhengerin (*n* = 3) (H&E). Normal mice receiving umuhengerin (group II) showed a normal histological structure of different neurons in the (**A**) cerebral cortex, (**B**) hippocampus and (**C**) striatum.

**Figure 8:**
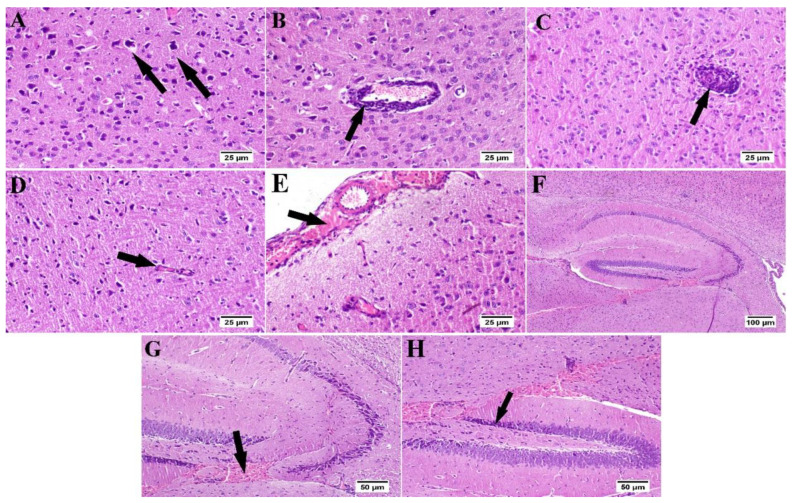
Histopathological alterations induced by STZ injection (*n* = 3) (H&E). Mice injected with STZ (group III) showed (**A**) dark degenerated neurons (arrows) of the cerebral cortex with neuronophagia and diffuse gliosis. The cerebral cortical blood vessels also exhibited (**B**) vasculitis (arrow) associated with neuronal degeneration and neuronophagia. (**C**) The striatum revealed severe vasculitis (arrow) with diffuse gliosis in addition to (**D**) congestion (arrow) and dark shrunken degenerated neurons. (**E**) The meninges showed congestion, thickening of the blood vessel wall, and perivascular hemorrhage (arrow). (**F**) The hippocampus revealed hemorrhage (arrow) with (**G**) dark shrunken degenerated neurons (arrow) in cornu ammonis (CA) 3 and 4 and (**H**) dentate gyrus (DG) regions.

**Figure 9:**
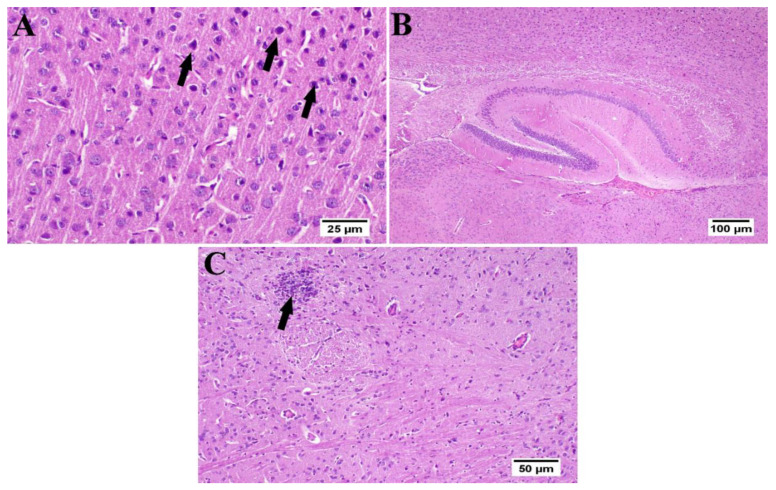
Effect of umuhengerin administration on mice brain histopathological alterations owing to STZ administration (group IV) (*n* = 3) (H&E). (**A**) The cerebral cortex showed few dark neurons (arrows). (**B**) The hippocampus showed apparently normal neurons in all examined regions. (**C**) The striatum revealed focal gliosis (arrow) and mild congestion.

**Figure 10:**
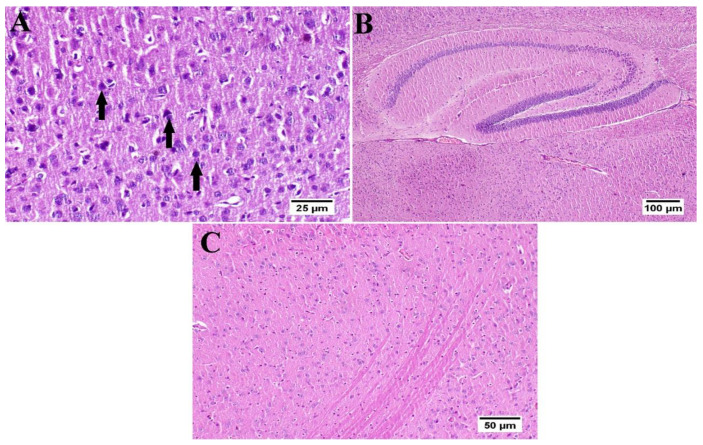
Effect of donepezil on STZ-injected mice brain histopathological examination (*n* = 3) (H&E). Animals treated with donepezil in group V showed (**A**) few degenerated neurons and neuronophagia (arrows) in the cerebral cortex. (**B**) The hippocampus and (**C**) striatum showed apparently normal neurons on all examined sites.

The photomicrographs of the cerebral cortex, hippocampus, and striatum of the control group (group I) revealed a normal structure of the brain tissue (Figure 6). Umuhengerin injection in group II did not alter the photomicrographs of the brain tissue, as compared with control animals (Figure 7). The microscopic examination of group III, which received STZ injection, showed numerous histopathological variations in the brain tissues. The cerebral cortex revealed multiple dark scattered degenerated neurons consorted with diffuse gliosis and neuronophagia. The blood vessels of the cerebral cortex also suffered from severe vasculitis in addition to diffuse gliosis in the striatum with dark degenerated neurons and congestion. The meninges exhibited marked congestion with thickening of the blood vessel wall and perivascular hemorrhage. Multifocal hemorrhagic areas with dark degenerated neurons were detected in the hippocampal cornu ammonis (CA) 3 and 4 as well as dentate gyrus (DG) regions (Figure 8). Umuhengerin administration in group IV ameliorated the toxic effect of STZ as only few cerebral cortex sections showed diminished numbers of dark neurons. The striatum showed focal gliosis with mild congestion of blood vessels. The hippocampus showed normal neurons (Figure 9). Mice treated with the standard drug (donepezil) in group V also showed normal cerebral cortex histological structure, except for neuronophagia and a few degenerated neurons. The striatum and the hippocampus appeared normal (Figure 10).

### 3.7. Effect of Umuhengerin Administration on Neuronal Survival Rate

**Figure 11:**
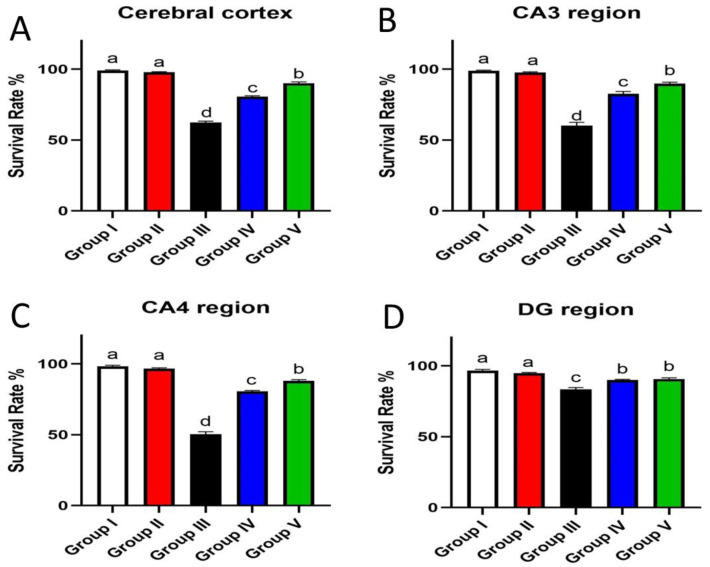
Effect of administration of umuhengerin on neuronal survival rate. Neuronal survival rate of different experimental groups was estimated in (**A**) the cerebral cortex, (**B**) CA3, (**C**) CA4, and (**D**) DG regions. Group I (control), group II (umuhengerin), group III (STZ), group IV (STZ + umuhengerin), and group V (STZ + donepezil). Values are expressed as mean ± SEM; *n* = 3. Different letters (a–d) above the error bar indicate statistically significant differences at *p* < 0.05.

**Figure 12:**
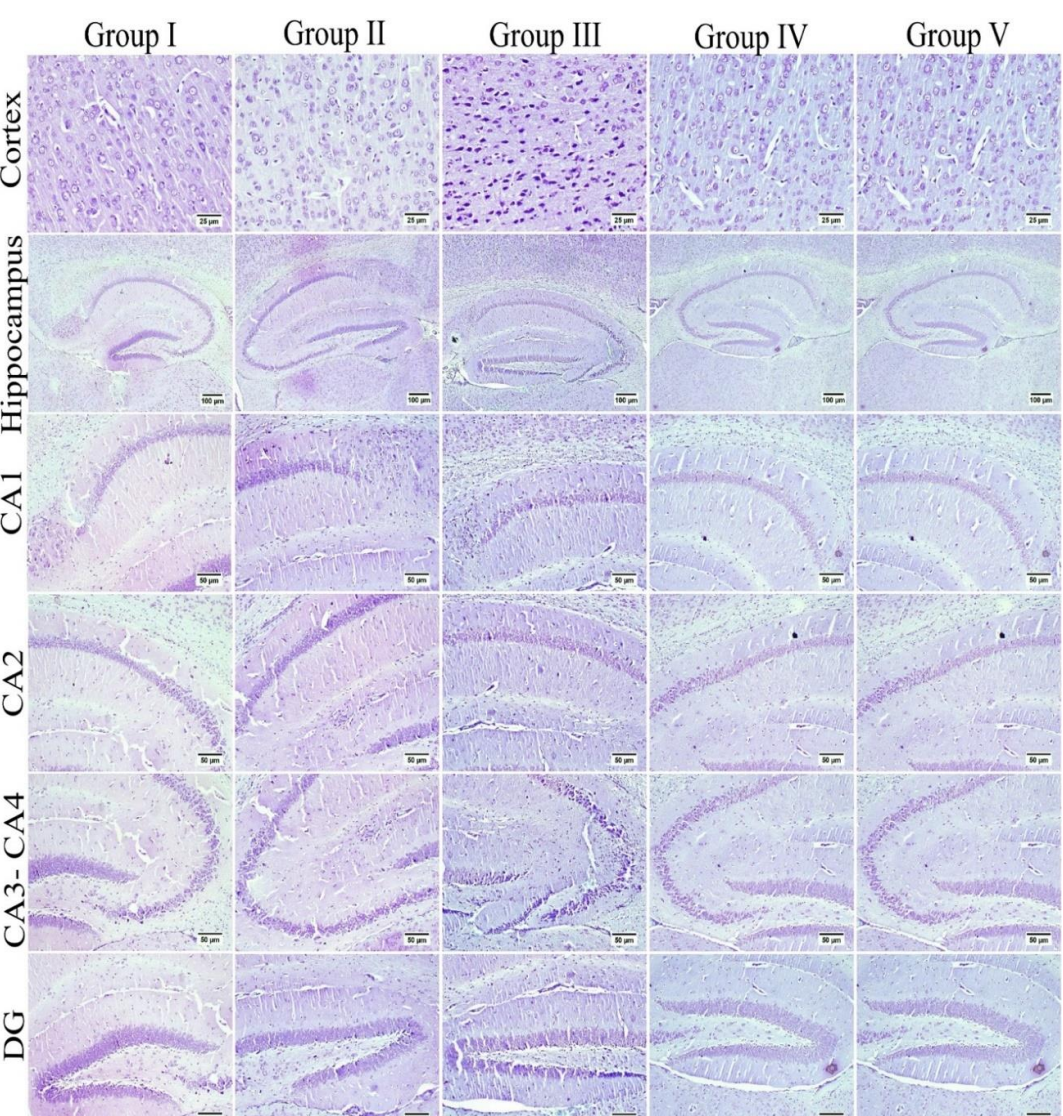
The neuronal survival rate was examined in different experimental groups using Nissl stain. The survival rate was inspected in the cerebral cortex and the hippocampal regions (CA3, CA4, and DG), which showed variation among different groups. Normal control mice and those which received umuhengerin (group I and II) showed normal intact neurons in all brain regions. An increased number of dark and shrunken neurons with pyknotic nuclei was detected in group III, which was injected with STZ. Marked preservation of neurons was observed in the treated groups whether receiving umuhengerin (group IV) or donepezil (group V).

The neuronal survival rate, which was examined in the cerebral cortex and the hippocampal regions (CA3, CA4, and DG), showed variation among the different groups. Normal control animals and those which received umuhengerin (group I and II, respectively) showed no significant difference in the survival rate. STZ-injected animals revealed a marked decrease in the neuronal survival rate, as compared with the control group. Umuhengerin-treated mice (group IV) displayed significant improvement in the neuronal survival rate in the cerebral cortex, CA3, CA4, and DG regions as compared to group III, which was injected with STZ alone. Donepezil-treated mice showed a significantly higher survival rate than umuhengerin-treated animals, except in the DG hippocampal region where both groups showed no significant difference. The affected neurons appeared shrunken, dark, and degenerated in the different brain regions (Figure 11 and Figure 12).

### 3.8. Effect of Umuhengerin Administration on Amyloid Plaques Numbers

**Figure 13:**
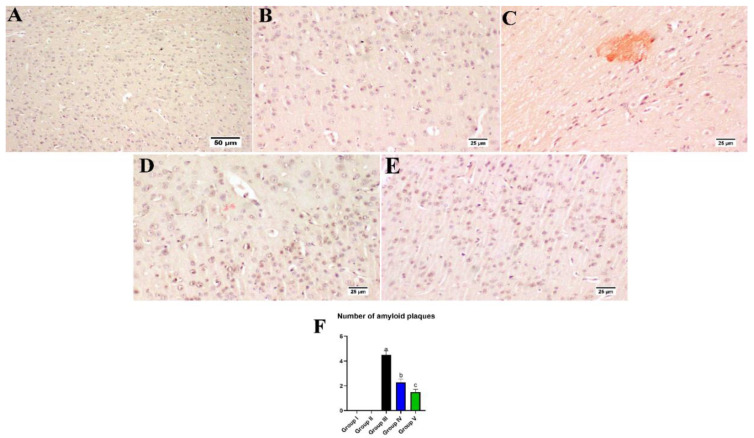
Effect of administration of umuhengerin on amyloid plaques number. Congo red stain was used for detection of amyloid plaques. Normal control mice showed an absence of amyloid deposition in the brain tissue whether in the (**A**) control group or (**B**) those injected with umuhengerin (groups I and II). (**C**) Amyloid deposition in the brain tissue were detected in group III, which received STZ injection. (**D**) Administration of umuhengerin resulted in a marked reduction in the number and size of amyloid plaques in the brain tissue. (**E**) Group V, which was injected with donepezil, showed few to an absence of amyloid plaques in most examined brain tissue sections. (**F**) Chart showing the number of amyloid plaques (high microscopic field) in different experimental groups. Values are expressed as mean ± SEM; *n* = 3. Different letters (a–c) above the error bar indicate statistically significant differences at *p* < 0.05.

Normal mice, whether injected with umuhengerin or not (group II or I, respectively), demonstrated no deposition of amyloid in the brain sections. Meanwhile, group III, injected with STZ, revealed an obvious amyloid deposition in the brain tissue. Umuhengerin administration resulted in a marked diminution in the number and size of amyloid plaques in the brain tissue in group IV. Group V, which was treated with donepezil, also showed almost no amyloid plaques in most of the examined regions (Figure 13).

## 4. Discussion

Flavonoids have been reported to retard the process of neurodegeneration [26]. Hence, the current work was aimed at determining umuhengerin flavonoid potential neuroprotective effects in STZ-injected mice. Administration of a subdiabetogenic STZ dose is a consistent model of sporadic AD (SAD), revealing many features of SAD, such as elevated oxidative stress and neuroinflammatory markers, as well as cholinergic deficits, accumulation of amyloid-beta (Aβ) proteins, memory, and learning derangement [27]. In the Morris water maze test, a significant increase in MEL was recorded in STZ-administered mice, in contrast to the decline in time spent in the target quadrant. This result is compatible with previous studies that reported that STZ injection is involved in learning and spatial memory deficits reflected in the compromised acquisition in the MWM task [22]. Treatment with either umuhengerin or the reference or positive control drug donepezil revealed significant spatial memory improvement shown as a marked reduction in MEL, and an evident increase in the time spent in the target quadrant on MWM trials and tests, respectively. Oxidative stress and neuroinflammation are substantial hallmarks of AD. The Nrf2 pathway, which is an endogenous defensive system capable of overcoming neuroinflammation and oxidative stress-induced pathologies associated with AD [28] is known to control the expression of glutathione-related enzymes, which play a vital role in preserving the redox state. Moreover, Nrf2 induces HO-1, which reduces superoxide and other free radicals’ generation; thus, it serves to defend the neurons against oxidative stress-induced toxicity [28]. Consistent with these findings, STZ-injected animals displayed a significant increase in the brain content of MDA, a marker of lipid peroxidation, and H_2_O_2_ free radical, which contrast with a prominent decrease in antioxidant defense systems, namely, GSH and HO-1 contents owing to decreased Nrf2 activity associated with upregulation of its inhibitor; Keap-1. Treatment with either umuhengerin or donepezil attenuated STZ-induced oxidative stress with increased Nrf2 activity in consequence of its associated protein Keap-1 downregulation, as well as elevated GSH and HO-1 contents. These observations are in contrast with the decreased MDA and H_2_O_2_ contents in the treated -STZ groups. Umuhengerin administration to normal mice exhibited no remarkable difference in the above markers, as compared with control animals.

The activation of Nrf2 is reported to inhibit inflammation by enhancing HO-1 expression and downregulating the NF-kβ signaling pathway which contributes to diverse inflammatory cytokines production, such as TNF-α [29]. The NF-kβ p65 level is reported to be significantly higher in the brains of AD patients [30]. TNF-α is implicated in the induction of phosphorylation and consequent degradation of the NF-Kβ inhibitor IkBα protein, thus activating NF-Kβ and promoting its translocation to the nucleus where it induces proinflammatory markers generation [31]. Moreover, proinflammatory cytokines have been connected to the degeneration of cholinergic basal forebrain cells and the induction of AChE activity leading to memory deficits due to impaired cholinergic transmission [32]. In AD, deposition of Aβ has been linked to NF-Kβ-induced upregulation of the β-secretase enzyme which supports the amyloidogenic pathway of amyloid precursor protein cleavage [31]. Consequently, in the current investigation, the STZ group exhibited prominent upregulation of β-secretase and a marked increase in AChE enzyme activity, with a subsequent increase in Aβ formation and cognitive decline, respectively. This was reflected histopathologically as an evident amyloid deposition in the brain tissues of this group. These pathological changes could be attributed to STZ-mediated neuroinflammation with increased TNF-α content and NF-Kβp65 upregulation along with its inhibitor; IKβα decreased expression. Administration of either umuhengerin or donepezil reversed STZ-induced inflammatory status with a consequent decrease in AChE activity and β-secretase protein expression owing to the downregulation of NF-Kβp65 in contrast to the increased expression of IKβα together with reduced TNF-α content. The favorable results achieved by umuhengerin are similar to donepezil. Regarding Aβ deposition, few or nearly absent Aβ plaques were detected in the donepezil-treated group, whereas the umuhengerin-treated group also revealed significant reduction in Aβ deposition.

STZ injection was previously linked to neuronal death [20]. Similarly, in the present study, the STZ group revealed a marked decrease in the neuronal survival percentage with plenty of shrunken dark and degenerated neurons in different brain regions. Administration of umuhengerin or donepezil enhanced neuronal survival in the hippocampal cornu ammonis (CA) 3 and 4 and cerebral cortex, as well as DG (dentate gyrus) regions. The STZ group showed various histopathological changes in the brain tissue with the cerebral cortex showing multiple dark scattered degenerated neurons that were accompanied with diffuse gliosis and neuronophagia. The blood vessels of the cerebral cortex suffered from severe vasculitis, in addition to diffuse gliosis in the striatum with dark degenerated neurons and congestion. The meninges exhibited marked congestion with thickening of the blood vessel wall and perivascular hemorrhage. Multifocal hemorrhagic areas with dark degenerated neurons in CA-3, CA-4, and DG regions were observed in the hippocampus. Treatment with umuhengerin ameliorated STZ deleterious effects as only few sections of the cerebral cortex exhibited reduced numbers of dark neurons with apparent healthy neurons in most of the investigated sections. The striatum showed focal gliosis with mild congestion of blood vessels, whereas the hippocampus showed apparently normal neurons. Mice treated with donepezil revealed a normal histological structure of the cerebral cortex except for neuronophagia and a few degenerated neurons, whereas the striatum and the hippocampus were apparently normal. Umuhengerin injection in the normal animals induced no histopathological changes like the normal control group.

## 5. Conclusions

Umuhengerin showed a marked improvement in oxidative stress and neuroinflammation associated with AD, along with an enhancement of cognitive abilities and neuronal survival owing to the retardation of Aβ aggregation. Umuhengerin-induced neuroprotective effects could be attributed to its ability to modulate Nrf2 and NF-Kβ signaling pathways. Therefore, umuhengerin is suggested as a potential candidate for AD management.

## Data Availability

Data is contained within the article and Supplementary Materials.

## Supplementary Materials

The following are available online at https://www.mdpi.com/article/10.3390/antiox10122011/s1, Western blot analysis details.
