# Supplementary material for: Umuhengerin Neuroprotective Effects in Streptozotocin-Induced Alzheimer’s Disease Mouse Model via Targeting Nrf2 and NF-Kβ Signaling Cascades
Source: Antioxidants (Basel). 2021 Dec 18;10(12):2011. doi: 10.3390/antiox10122011 (PMC8698785; doi:10.3390/antiox10122011)
Supplement: Supplementary file 1 [file antioxidants-10-02011-s001.zip › antioxidants-1494252-supplementary.pdf]

Supplementary data

Article

Umuhengerin neuro-protective effects in streptozotocin-induced Alzheimer's disease mouse model via targeting Nrf2 and NF-Kβ signaling cascades

Alaa Sirwi [1], Nesrine S. EL Sayed [2], Hossam M. Abdallah [1,3*], Sabrin R. M. Ibrahim [4,5], Gamal A. Mohamed [1,6], Ali M. El-Halawany [3], Martin K. Safo [7], Nora O. Abdel Rasheed [2]

[1] Department of Natural Products and Alternative Medicine, Faculty of Pharmacy, King Abdulaziz University, Jeddah, Saudi Arabia; hmafifi@kau.edu.sa, asirwi@kau.edu.sa (A.S.); gahussein@kau.edu.sa (G.A.M.)
[2] Department of Pharmacology and Toxicology, Faculty of Pharmacy, Cairo University, Giza 11562, Egypt
[3] Department of Pharmacognosy, Faculty of Pharmacy, Cairo University, Giza 11562, Egypt
[4] Batterjee Medical College, Preparatory Year Program, Jeddah 21442, Saudi Arabia (sabrin.ibrahim@bmc.edu.sa)
[5] Department of Pharmacognosy, Faculty of Pharmacy, Assiut University, Assiut 71526, Egypt
[6] Faculty of Pharmacy, Pharmacognosy Department, Al-Azhar University, Assiut Branch, Assiut 71524, Egypt
[7] Department of Medicinal Chemistry, Institute for Structural Biology, Drug Discovery and Development, School of Pharmacy, Virginia Commonwealth University, Virginia, 23219, USA
*Correspondence: hmafifi@kau.edu.sa; Tel.: 00966544733110

Western blot analysis

The study was performed for estimating Nrf2, Keap-1, β-secretase, NF-Kβ-p65, as well as its inhibitor IKβα activities. The procedure was carried out as previously reported by [20] with the nitro-cellulose membranes being incubated with 1:1000 dilutions of the following primary antibodies: Nrf2, β-secretase, NF-Kβ p65, Keap-1, and IKβα (Cell Signaling Technology, USA). Afterwards, they were probed with the peroxidase-labelled secondary antibodies with 1:5000 dilution(Dianova, Germany). Lastly, the band intensities were specified by densitometric analysis using a scanning laser densitometer. Results were displayed as arbitrary units relative to the corresponding β-actin band intensity.

Detailed procedure of Western blot

After the behavioral tests were carried out, brains were removed for Western blot analysis. Western blot is a method to quantify expression level of specific proteins. After protein solutions are extracted from tissues, the protein concentration can be easily determined; equal amounts of proteins are loaded onto a sodium dodecyl sulfate polyacrylamide gel electrophoresis (SDS-PAGE). SDS-PAGE allows separation of proteins according to their molecular weight. Transfer of proteins (blotting) to a nitrocellulose membrane

or polyvinylidene fluoride (PVDF) is necessary for subsequent antibody detection of the proteins of interest. Membranes are then incubated with a specific antibody recognizing the target protein, and subsequently with a secondary antibody, which is horseradish peroxidase-conjugated. Incubation with the substrate permits the detection of the amount of protein through optical documentation systems. The quantification of the band intensity can be used to determine specific protein levels in the tested tissues. Detection of a second “housekeeping” protein is necessary to control for variability in protein loading between samples.

Reagents

1. Radio immunoprecipitation assay (RIPA) buffer: This was prepared by mixing 1.5 ml 5M NaCl, 1mM phenylmethylsulfonyl fluoride (MW 174.19), 2.5 ml 10% deoxycholic acid (DOC), 0.5 ml 10% SDS, 2.5 ml 1M Tris (pH 8.6) and the final volume was completed to 50 ml with deionized water.
2. Laemmli buffer for SDS-PAGE: It was prepared by dissolving 0.757 g Tris-base,10 mg bromophenol blue and 6 g SDS in 20 ml glycerol and the volume was completed to 90 ml with deionized water and 10 ml of β-mercaptoethanol was added prior use immediately.
3. Tris buffered saline tween (TBST) buffer: It was prepared by dissolving 12.1 g Tris, 40 g NaCl in 20.25 ml tween 20 and deionized water was added to complete a final volume of 5 l and pH was adjusted to 7.56.
4. Ponceau solution: It was prepared by dissolving 0.5 g Ponceau S (0.5%) in 1 ml glacial acetic acid and the final volume was completed to 100 ml with deionized water.
5. Blocking solution: This was prepared by dissolving 6 g non-fat dry milk in 100 ml TBST buffer.
6. Electrophoresis buffer: It was prepared by dissolving 30.29 g Trisbase, 144.13 g glycine and 10 g SDS in deionized water and the final volume was completed to 1 l.
7. Transfer buffer: It was prepared by dissolving 12.1 g Tris-base, 56.3 g glycine in 1000 ml methanol and the final volume was completed to 5 l with deionized water.

Procedures

1. Sample preparation and determination of protein concentration. About 100 mg of tissue was homogenized with 150 µl RIPA buffer using a T-10 Basic Ultra Turrax homogenizer (IKA-Werke GmbH & Co., USA). All steps were performed on ice to avoid enzymatic degradation of proteins. The homogeneous lysates were transferred into a micro-centrifuge and spun at 12,000 rpm at 4 °C for 20 min. After centrifugation, the lysate was then collected and protein concentration was determined with a Bicinchoninic acid protein assay (BCA) kit (Thermo Fisher Scientific Inc., USA).

2. Electrophoresis, samples loading and gel running. To separate proteins according to their molecular weight, it is necessary to denature proteins with an anionic detergent SDS. Since SDS adds a negative charge to denatured proteins, the protein's own charge is masked so that proteins can be separated according to their molecular weight. An aliquot of 7.5 μg protein of each sample was boiled with Laemmli buffer at 95 °C for 5 min to ensure that proteins were denatured. Each sample was then loaded into an individual lane in SDS-PAGE and a marker protein of known molecular weight was loaded also into separate lane to estimate the molecular weight of the proteins of interest to be detected in the protein samples under investigation. Gels were run at 50–120 V in an electrophoresis chamber using electrophoresis buffer until the blue marker (Laemmli buffer) reached the bottom of the gel.
3. Transfer of proteins and staining (western blotting). Two fiber pads and two Whatman filter papers were soaked in pre-cooled transfer buffer (4 °C). The polyacrylamide gel was transferred onto a soaked Whatman filter paper and the nitrocellulose membrane was placed gently on top of the gel and then covered with the second Whatman filter paper. The sandwich was then placed between the pre-soaked fiber pads. The gel sandwich was put into a cassette which was introduced into the Mini Trans-Blot Cell apparatus. Proteins were transferred at a current of 30 V overnight at 4 °C. Negatively charged proteins moved and adhered onto the membrane while maintaining the size specific organization they had within the gel. The nitrocellulose membrane was stained in a Ponceau solution for 30 s to check uniform transfer of proteins. The membrane was then washed several times in TBST buffer to remove Ponceau dye before proceeding to immunostaining of membranes with antibodies and detection of signals with colour reagents. The membrane was blocked with a 6% blocking solution containing non-fat dry milk in TBST buffer for 3 h at 4 °C to avoid background signals due to non-specific interactions of the respective antibodies with the nitrocellulose membrane. The membranes were then washed with TBST buffer and incubated with 1:1000 dilutions of the following primary antibodies: Nrf2(#12721), β-secretase(#5606), NF-Kβ p65(#6956), Keap-1(#4678), IKβα (#4814), and β=actin (#3700) (Cell Signaling Technology, USA) overnight at 4 °C .The membranes were then washed 5 times for 5 min each in TBST to remove unbound primary antibody. Afterwards, they were probed with the peroxidase labelled secondary antibodies with 1:5000 dilution(Dianova, Germany) at room temperature for 1 h. The membranes were again washed 5 times for 5 min each in TBST buffer. Lastly, the band intensities were specified by densitometric analysis using a scanning densitometer. Results were displayed as arbitrary units relative to the corresponding β-actin band intensity.
